# Long noncoding RNA GAS5 inhibits progression of colorectal cancer by interacting with and triggering YAP phosphorylation and degradation and is negatively regulated by the m^6^A reader YTHDF3

**DOI:** 10.1186/s12943-019-1079-y

**Published:** 2019-10-16

**Authors:** Wen Ni, Su Yao, Yunxia Zhou, Yuanyuan Liu, Piao Huang, Aijun Zhou, Jingwen Liu, Liheng Che, Jianming Li

**Affiliations:** 1 Department of Pathology, Sun Yat-sen Memorial Hospital, Sun Yat-sen University, Guangzhou, 510120 China; 2 Guangdong Provincial Key Laboratory of Malignant Tumor Epigenetics and Gene Regulation, Sun Yat-sen Memorial Hospital, Sun Yat-sen University, Guangzhou, 510120 China; 3 RNA Biomedical Institute, Sun Yat-sen Memorial Hospital, Sun Yat-sen University, Guangzhou, 510120 China

**Keywords:** LncRNA GAS5, YAP signaling, YTHDF3, Ubiquitination and degradation, m^6^A modification

## Abstract

**Background:**

YAP activation is crucial for cancer development including colorectal cancer (CRC). Nevertheless, it remains unclear whether N6-Methyladenosine (m^6^A) modified transcripts of long noncoding RNAs (lncRNAs) can regulate YAP activation in cancer progression. We investigated the functional link between lncRNAs and the m^6^A modification in YAP signaling and CRC progression.

**Methods:**

YAP interacting lncRNAs were screened by RIP-sequencing, RNA FISH and immunofluorescence co-staining assays. Interaction between YAP and lncRNA GAS5 was studied by biochemical methods. MeRIP-sequencing combined with lncRNA-sequencing were used to identify the m^6^A modified targets of YTHDF3 in CRC. Gain-of-function and Loss-of-function analysis were performed to measure the function of GAS5-YAP-YTHDF3 axis in CRC progression in vitro and in vivo.

**Results:**

GAS5 directly interacts with WW domain of YAP to facilitate translocation of endogenous YAP from the nucleus to the cytoplasm and promotes phosphorylation and subsequently ubiquitin-mediated degradation of YAP to inhibit CRC progression in vitro and in vivo. Notably, we demonstrate the m^6^A reader YTHDF3 not only a novel target of YAP but also a key player in YAP signaling by facilitating m^6^A-modified lncRNA GAS5 degradation, which profile a new insight into CRC progression. Clinically, lncRNA GAS5 expressions is negatively correlated with YAP and YTHDF3 protein levels in tumors from CRC patients.

**Conclusions:**

Our study uncovers a negative functional loop of lncRNA GAS5-YAP-YTHDF3 axis, and identifies a new mechanism for m^6^A-induced decay of GAS5 on YAP signaling in progression of CRC which may offer a promising approach for CRC treatment.

## Introduction

Colorectal cancer (CRC) is one of the leading cancer types resulting in new cancer cases and deaths worldwide [1]. Increasing evidence, including ours, shows that dysregulation of Hippo/YAP signaling contributes to tumorigenesis, including CRC [2, 3]. YAP drives target gene expression by forming complexes with multiple transcription factors, which are required to drive tumor initiation and progression [4]. Phosphorylation of YAP, a major downstream transducer of the Hippo pathway [5], is a key event in YAP signaling. Both cytoplasmic and nuclear localization of YAP could be regulated by YAP phosphorylation at different sites by different kinases [6, 7]. In addition to these, lysine methylation is another important post-translational modification involved in YAP activation and location [8]. However, the new factors and the concise mechanisms for regulation of subcellular localization and activation of YAP are still poorly known.

Long non-coding RNAs (lncRNAs) are transcripts longer than 200 nucleotides that have no or limited protein-coding capacity. A large body of evidence has demonstrated that lncRNAs are engaged in the signaling pathways of CRC. LncRNAs are important versatile molecules involved in a variety of tumorigenic processes and diseases via interactions with DNA, RNA, or proteins. LncRNAs execute molecular functions as archetypes of decoys, signals, guides, and scaffolds [9]. For instance, XIST is one of first functionally annotated lncRNAs that plays a critical role in X inactivation by recruiting multiple factors [10]. HOTAIR is a lncRNA of the HOXC locus, which forms RNA-DNA-DNA triplexes with predicted target sites in mesenchymal stem cells [11]. LncRNA nuclear enriched abundant transcript 1 (NEAT1) has a profound effect on cross-regulation between paraspeckles and mitochondria by altering the sequestration of mito-mRNAs in paraspeckles [12]. LncRNA growth arrest-specific 5 (GAS5) induces apoptosis by binding to the domain of the glucocorticoid receptor [13]. Recently, we found that lncRNA uc.134 inhibits YAP downstream target genes by inhibiting CUL4A to ubiquitinate LATS1 and increasing pYAP^S127^ expression [14]. Nevertheless, whether lncRNAs can regulate YAP activation by direct interaction or post-translationally modifying YAP protein remains to be elucidated.

The N6-methyladenosine (m^6^A) RNA modification, as the most abundant internal epi-transcriptomic modification in eukaryotic messenger RNAs (mRNAs), is introduced by the m^6^A methyltransferase complex, which have been designated as a “writer,” and can be deleted by m^6^A demethylases, such as fat mass and obesity-associated protein (FTO) and ALKBH5. Factors interpreting specific modifications have been identified as “readers,” such as YTHDF1/2/3 and YTHDC1/2 [15]. FTO is the first m^6^A demethylase that is highly expressed in acute myeloid leukemia (AML), and it plays a critical oncogenic role [16]. YTHDF3 facilitates translation of protein synthesis in synergy with YTHDF1 and affects decay of methylated mRNA mediated through YTHDF2 [17]. Although the m^6^A is reported to be important in cancer progression, whether lncRNAs regulate the m^6^A modification and the role of m^6^A in lncRNA transcripts in cancer progression remain unknown. Here, we aimed to investigate the functional links between lncRNAs and the m^6^A modification in YAP signaling in CRC.

## Materials and methods

### Cell lines, cell culture, and transfection

DLD1, LOVO, SW480, SW620, LS174T, HCT116, RKO, and HT29 cell lines were obtained from the Cell Bank of Type Culture Collection (Guangzhou Cellcook Biotech Co., Ltd., Guangzhou, China). The cell line authentication report showed the cell lines to be considered as identical to the reference cell line in the ATCC STR database. The cells were cultured in incubators containing 5% CO_2_ at 37 °C and were maintained in RPMI 1640 supplemented with 10% FBS. The transfection of plasmids or small interfering RNAs (siRNAs) was performed using jetPRIME (Polyplus, Strasbourg, France), according to the manufacturer’s instructions. As we previously described, 24 h after transfection, cell lysates were subjected to western blot, and the western blot data were quantified using the ImageJ software [14]. Detailed descriptions of antibodies, oligonucleotide sequences and primers can be found in the Additional file 2.

### RIP sequencing

An RIP experiment was performed according to the instructions of the Magna RIP RNA Binding Protein Immunoprecipitation Kit (Millipore, MA, USA). Briefly, lysate was prepared in a lysis buffer containing protease inhibitor cocktail and RNase inhibitor. Then, protein A/G magnetic beads were prepared for incubation with 5 μg of purified antibodies per immunoprecipitation with rotation for 30 min at room temperature. Further, to precipitate RNA-binding protein-RNA complexes, and the mixture was incubated with rotation for 3 h overnight at 4 °C. Finally, RNA was purified using proteinase K buffer and an Agilent 2100 Bioanalyzer (Agilent, CA, USA). A NanoDrop 2000 (Thermo Fisher, MA, USA) was used to analyze the total RNA quality and quantity. Further RNA purification in an immunoprecipitation may be pursued by deep sequencing or quantitative reverse transcription polymerase chain reaction (qRT-PCR). The cDNA libraries were sequenced on the Illumina sequencing platform by Genedenovo Biotechnology Co., Ltd. (Guangzhou, China).

### MeRIP sequencing

Total RNA was extracted using Trizol reagent (Takara, Dalian, China). An Agilent 2100 Bioanalyzer (Agilent, CA, USA) and NanoDrop 2000 (Thermo Fisher, MA, USA) were used to analyze the total RNA quality and quantity. More than 50 μg of total RNA is sufficient following RNA fragmentation and immunoprecipitation according to the instructions of the Magna MeRIP™ m^6^A Kit (Merck, Darmstadt, Germany). Briefly, the total cell RNA is fragmented into ~ 100-nt-long oligonucleotides using fragmentation buffer under elevated temperature. Then the post-fragmentation size distribution is validated by an Agilent 2100 Bioanalyzer with an Agilent RNA 6000 Kit. The Magna ChIP Protein A/G Magnetic Beads were incubatated for 30 min at room temperature with m^6^A-specific antibody in immunoprecipitation buffer. The mixture was then incubated with the MeRIP reaction mixture for 2 h at 4 °C. Then eluted RNA and MeRIPed RNA were analyzed by deep sequencing on an Illumina Novaseq™ 6000 platform at the LC-BIO Bio-tech ltd (Hangzhou, China) following the vendor’s recommended protocol.

#### In vivo model

BALB/c male mice 6–8 weeks old were purchased from Guangdong Medical Laboratory Animal Center, China. Mice were raised under pathogen-free conditions. All in vivo experiments were done according to approved protocols from the Institutional Animal Care and Use Committees, according to national and institutional guidelines. All procedures were performed essentially as previously described. Briefly, for the subcutaneously injected tumor model, 2 × 10^6^ viable cells were subcutaneously injected into the flanks of mice. Tumor volume was assessed as (L × W^2^/2), where L and W represent the length and the width of the tumor, respectively. After 4 weeks, the tumors were embedded in paraffin and stained for in situ hybridization (ISH) or immunohistochemistry (IHC). For the lung metastasis model, 2 × 10^6^ viable cells were injected into the tail veins of mice. The mice were monitored for 6 weeks for lung metastasis by hematoxylin-eosin (H&E) staining. The metastatic foci were calculated using Dmetrix software by combining the number and area of lung metastatic nodules in individual mice.

#### Tissue samples, immunohistochemistry (IHC), and in situ hybridization (ISH) staining

Formalin-fixed paraffin-embedded (FFPE) colon cancer tissues and adjacent noncancerous tissues were retrieved from the Department of Pathology at Sun Yat-sen Memorial Hospital, Sun Yat-sen University (Guangzhou, China). The study was approved by the Human Research Ethics Committees of Sun Yat-sen University. The study is compliant with all relevant ethical regulations for human research participants, and informed consent was obtained from all subjects.

All procedures were performed essentially as previously described [14]. Briefly, for H&E staining, sections of tissue were deparaffinized in xylene and then stained with hematoxylin and eosin according to standard histological procedures. For IHC staining, after the sections were deparaffinized and re-hydrated, the specimens were incubated in EDTA buffer (1 mM, PH 8.0) for antigen retrieval using a high-pressure method. Then, tissue sections were incubated overnight at 4 °C with primary antibodies, including anti-YAP, anti-YTHDF3, and anti-Ki 67. 3,3′-diaminobenzidine (DAB) solution (ZSGB-BIO, Beijing, China) was used to detect target proteins, which were conjugated with a peroxidase enzyme to form a brown precipitate. For ISH staining, lncRNA GAS5 expression was measured in paraffin-embedded samples according to the instructions of the ISH Kit™ (BOSTER, Wuhan, China). Briefly, after the sections were deparaffinized and re-hydrated, the specimens were incubated with proteinase for 10 min. at 37 °C. After washing twice in PBS, the hybridization mix was applied, and hybridized samples were incubated overnight at 40 °C. Then, the sections were incubated with blocking solution for 30 min and anti-DIG reagent was applied for 60 min. Then, the sections were incubated with AP substrate 4-nitro-blue tetrazolium and 5-bromo-4-chloro-3′-indolylphosphate (NBT-BCIP) for 30 min at 37 °C. The sections were mounted with Nuclear Fast Red. A blue stain in the samples indicated a positive signal by NBT-BCIP. For DAB-stained samples, a brown precipitate showed a positive signal, and the slides were then counterstained with hematoxylin.

The staining scores were evaluated by two individuals in a blinded fashion. A quick scoring system from 0 to 12 that combined the intensity and percentage of the positive signal was used as described previously [14, 18]. Briefly, a signal of 0 indicated no staining, 1 indicated weak staining, 2 indicated intermediate staining and 3 indicated strong staining. Percentage scores were assigned as follows: 0 corresponded to 0%, 1 to 1–25%, 2 to 26–50%, 3 to 51–75%, and 4 to > 75%. The median value of total staining scores was identified as the optimal cut-off value. If the evaluated score was lower than the median, the indicator expression of in those CRC samples was classified as low; otherwise, it was classified as high.

#### RNA-pulldown assay

Biotin-labeled RNAs were transcribed in vitro with the Biotin RNA Labeling Mix and T7 RNA polymerase (Roche, Basel, Switzerland). Biotinylated RNAs were mixed with streptavidin agarose beads (Life Technologies, Gaithersburg, MD) at 4 °C overnight. Total cell lysates were freshly prepared and added to each binding reaction with Protease/Phosphatase Inhibitor Cocktail and RNase inhibitor, and then the mixture was incubated with rotation for 1 h at 4 °C. After washing thoroughly three times, the RNA–protein binding mixture was boiled in SDS buffer and the eluted proteins were detected by western blot or mass spectrometry.

#### Bio-layer interferometry (BLI) analysis

A bio-layer interferometry (BLI) experiment was carried out using the Octet system (ForteBio, Fremont, CA). Streptavidin sensors were used for immobilization of biotin-labeled lncRNA GAS5. A five-point concentration series was assayed for purified YAP protein (10-nM to 1-μM range). Wells with assay buffer only were used as reference wells. Reference biosensors with no immobilized ligand was used to avoid nonspecific binding to GAS5 during the binding event. The procedure of Octet System Data Acquisition software was followed to analyze the kinetics. Dissociation (Kd) and association rate constants (Ka) were determined with the Octet Data Analysis Software, as a result of a global fit considering the entire step times, and assuming a 1:1 binding model.

#### RNA FISH and immunofluorescence co-staining

The locked nucleic acid-modified oligonucleotide probe targeting GAS5 (Exiqon, Vedbaek, Denmark) was used for RNA fluorescent in situ hybridization (FISH). We detected YAP protein in situ in CRC cells with immunofluorescence assays. The anti-YAP1 antibody (Alexa Fluor 647) (abcam, MA, USA) was used to detective the YAP protein with a confocal microscope (Leica, Wetzlar, Germany) (shown in red), and DAPI was used for labelling nuclear DNA (shown in blue). The RNA signal was detected by incubation with biotinylated conjugated anti-DIG antibodies, and the signals were amplified using SABC – FITC (shown in green).

#### Chromatin immunoprecipitation (ChIP) and luciferase reporter assay

The chromatin immunoprecipitation (ChIP) procedure was performed using SimpleChIP® Enzymatic Chromatin IP Kit (Cell Signaling Technology, MA, USA) following the manufacturer’s instructions. Briefly, cells are fixed with formaldehyde to cross-link histone and non-histone proteins to DNA. Then, chromatin is digested with micrococcal nuclease into 150- to 900-bp DNA/protein fragments. Antibodies specific to YAP proteins are added and the complex co-precipitates are captured by Protein G magnetic beads. Finally, cross-links are reversed, and the level of enrichment of the target DNA sequence is purified, at which point it is ready for PCR. One tenth of the input chromatin was also treated in the same way and purified. The enriched DNA fragments were presented as a percentage of input chromatin. Luciferase reporter assay was measured using the Dual-Glo Luciferase Assay System (Promega, WI, USA) following the manufacturer’s instructions. Briefly, YAP plasmids were co-transfected with YTHDF3 promoter-luciferase vector and pRL-TK Vector. The pGL3-basic vector was transfected as a negative control. After 24 h, prepared cell extracts were used to measure the luciferase activity on a Spark multimode microplate reader (TECAN, Mannedorf, Switzerland).

#### Quantification and statistical analysis

All statistical analysis was carried out using GraphPad Prism version 7 (GraphPad Software, CA) for Windows to assess the differences between experimental groups. The data were analyzed by analysis of variance tests or Student’s t-tests (*P < 0.05, **P < 0.01, ***P < 0.001). A multi-way classification analysis of variance tests was performed to assess data obtained from the CCK8 assays and tumor growth. Survival curves were plotted based on the Kaplan–Meier curves and log-rank tests. Correlations among GAS5 expression, YAP, and YTHDF3 were analyzed with a Spearman rank correlation. *P <* 0.05 was considered to indicate a significant difference. Each experiment was repeated independently with similar results at least three times.

#### Data availability

The RIP-sequencing, lncRNA-sequencing, and MeRIP-sequencing data discussed in this paper have been deposited in NCBI’s Gene Expression Omnibus [19] and are accessible through GEO Series accession numbers GSE129535, GSE129624 and GSE129716. The data will become public when this article is published online (Additional files 4, 5 and 6).

## Results

### Screening and identification of YAP-interacting lncRNAs

**Fig. 1:**
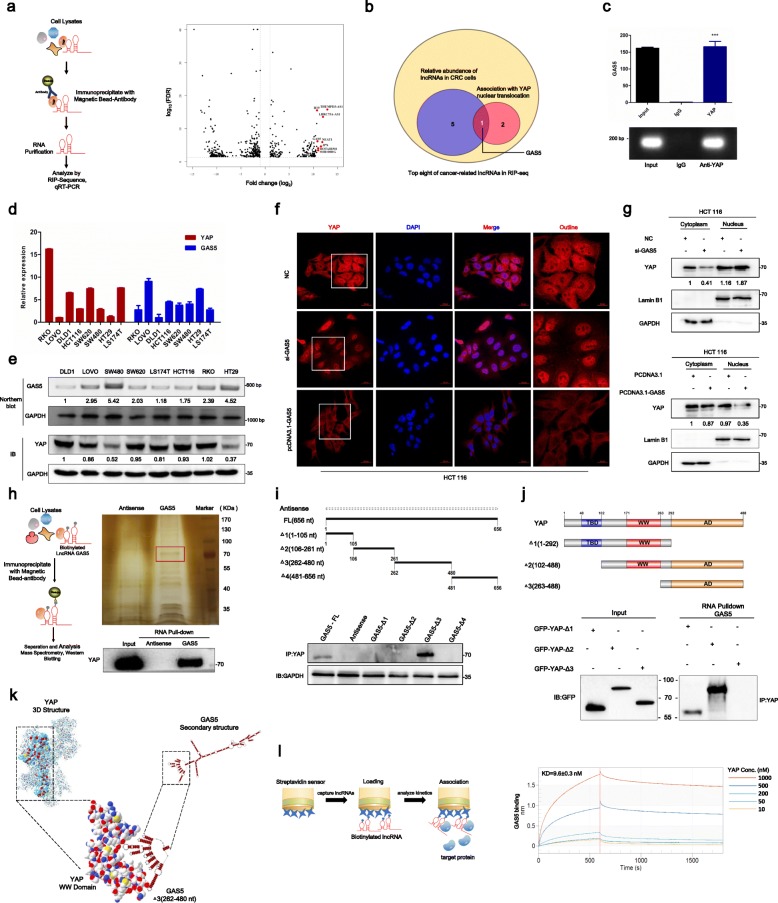
Screening and identification of YAP-interacting lncRNA GAS5. **a** RIP-seq experiments were performed to identify YAP-binding lncRNAs. Volcano plot showing the differentially expressed lncRNAs upon YAP immunoprecipitation. Red dots mark top eight upregulated lncRNAs (fold change > 2, FDR < 0.05). **b** Screening strategy was used to find key YAP-binding lncRNAs in colorectal cancer (CRC). **c** RIP assays for YAP were performed and the coprecipitated RNA was subjected to qRT-PCR for GAS5 (upper panel). Agarose electrophoresis of PCR products (bottom panel). Experiments were performed in triplicate, and data are presented as mean ± SD. ***P < 0.001. **d** The expression of YAP and GAS5 were analyzed by qRT-PCR in various CRC cell lines. **e** GAS5 and YAP expressions in various CRC cell lines, shown by northern blot and western blot (IB). **f-g** GAS5 facilities YAP cytoplasmic retention as demonstrated by immunofluorescence staining **(f)** and western blot **(g)**. **h** RNA pull-down assay (upper panel) and western blot assays (bottom panel) showed that biotinylated-GAS5 could bind with YAP in CRC cells in vitro. **i** Immunoblot (IB) detection of YAP, which were pulled down by in vitro transcribed biotinylated RNAs corresponding to different fragments of GAS5 in CRC cells. **j** IB detection of GFP-tagged YAP (WT versus domain truncation mutants) precipitated by in vitro transcribed biotinylated-GAS5 in HEK293T cells. Upper panel: graphic illustration of the domain structure of YAP. **k** Visualization of interaction between YAP, the 3D structure of which was shown, and GAS5, the secondary structure model of GAS5 was simulated by RNAstructure software. **l** Bio-layer interferometry (BLI) analysis of biotinylated-GAS5 binding to YAP protein

We initiated this study by screening YAP-interacting lncRNAs. The profile of the RIP-seq experiments identified a number of candidates for YAP-interacting lncRNAs (Fig. 1a). To validate our findings from the sequencing data and study their role in CRC, the top eight of over two-fold enrichment of cancer-related lncRNAs in the YAP-bound portion compared with IgG antibody using qRT-PCR and agarose gel electrophoresis were analyzed further for conservation across species, transcript abundance in CRC cells, and the effects on YAP nuclear translocation (Fig. 1b-g, Additional file 1: Figure S1). According to these characters, GAS5 was selected for our next study (Fig. 1b). We used 5′-and 3′-RACE analyses to identify a 656-bp full-length transcript of GAS5 in CRC cells (Additional file 1: Figure S2) and analyzed the expressions of selected lncRNAs and YAP in eight CRC cell lines by qRT-PCR (Fig. 1d-e; Additional file 1: Figure S1). Interestingly, GAS5 expression showed a negative correlation with YAP expression in CRC cells by qRT-PCR, northern blot, and western blot analysis (Fig. 1d-e).

The endogenous YAP nuclear localization is known to be paralleled by transcriptional activation of the target genes such as CTGF and CYR61 [2]. Phosphorylated YAP sequestered in the cytoplasm by facilitating its binding to 14–3-3 protein [20]. Yet, whether lncRNAs can directly regulate YAP activation remains unknown. Interestingly, immunofluorescence staining assay indicated that nuclear YAP protein accumulated when GAS5 was suppressed, whereas nuclear YAP protein decreased when GAS5 was overexpressed (Fig. 1f). Western blot using cytoplasmic and nuclear protein fractions isolated from CRC cells further showed that GAS5 inhibited the nuclear accumulation of YAP (Fig. 1g). Collectively, our study identified numbers of candidates for YAP-interacting lncRNAs and found that GAS5 interacts directly with YAP and inhibits nuclear accumulation of YAP.

### Biochemical characterization of interaction between GAS5 and YAP protein

We further performed RNA pull-down assays and subsequent western blot analyses to characterize how GAS5 directly binds to YAP (Fig. 1h). To identify the unique binding sites, we took advantage of a series of deletion mutants of GAS5 to map the YAP binding region (Fig. 1i). RNAfold and RNAstructure software were used to predict the secondary structure of GAS5, all deletion mutants of GAS5 mostly preserved the RNA hairpin structures (Additional file 1: Figure S4). Results showed GAS5 mutants Δ3 bound to YAP as efficiently as full-length GAS5, whereas other mutants completely lost their binding capacity (Fig. 1i), indicating that nucleotides 262–480 of GAS5 are required for the association with YAP. To identify the regions of YAP responsible for its binding with GAS5, we constructed a series of YAP domain-deletion mutants. Protein domain mapping studies demonstrated that GAS5 binds the 171–263 amino acid (aa) region of YAP (Fig. 1j). The 171–263 aa region of YAP encodes domains known as the WW domain, which has been suggested to bind proteins with particular proline motifs, such as [AP]-P-P-[AP]-Y. I-TASSER software from Zhang’s lab was used to predict the 3D structure of the YAP protein, and the C-score of the predicted model was − 1.05, which signifies model accuracy with a high degree of confidence. Then, we further used SWISS-MODEL and RNAstructure software to predict and analyze the 3D structure of the YAP protein WW domain and GAS5 RNA secondary structure, respectively. Figure 1k visualizes the structure of the YAP-GAS5 binding complex.

We next performed other two independent methods, including Bio-layer interferometry (BLI) analysis, RNA fluorescent in situ hybridization (RNA FISH) and immunofluorescence co-staining assay, to identify the interaction between lncRNA GAS5 and YAP. BLI analysis was performed to examine the binding affinity of YAP–GAS5 complex with a Fortebio Octet system. The illustration in Fig. 1l visualizes the procedure of the BLI kinetics experiment. Increased concentrations of YAP protein were used to test for dose-dependent changes in the BLI signal. The RNA binding affinity of YAP to the biotinylated lncRNA GAS5 is specific with a K_D_ of ∼9.6 nM (Fig. 1l). Furthermore, RNA FISH and immunofluorescence co-staining assays showed that YAP was mainly localized in the nucleus, while GAS5 was localized in both the cytoplasm and the nucleus but mainly in the cytoplasm. Further, co-localization of GAS5 and YAP in CRC cells strongly supports the binding of GAS5 with YAP inside cells. Exogenous expression of GAS5 could lead to translocation of endogenous YAP from the nucleus to the cytoplasm (Additional file 1: Figure S3). Taken together, biochemical mapping demonstrate that GAS5 directly interacts with the WW domain of YAP to facilitate translocation of endogenous YAP from the nucleus to the cytoplasm.

### GAS5 promotes YAP phosphorylation to facilitate its ubiquitination and degradation

**Fig. 2:**
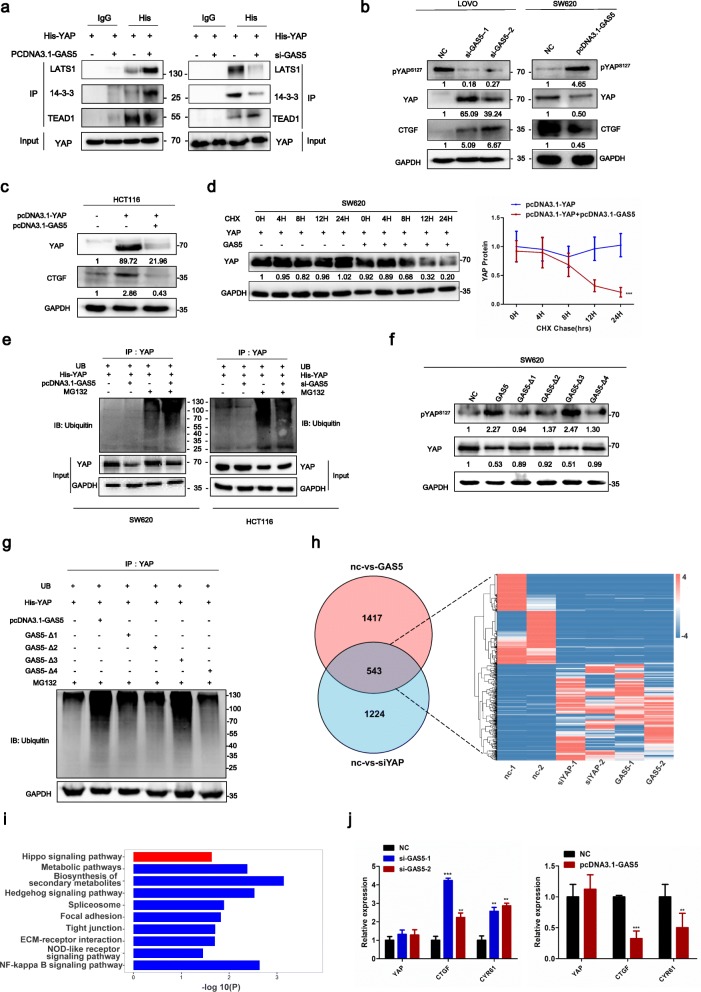
GAS5 facilitates YAP cytoplasmic retention and promotes phosphorylation and ubiquitination-mediated degradation of YAP. **a** HEK293T cells were co-transfected with the YAP and GAS5 plasmid or indicated siRNA, and the interaction between YAP and LATS1/14–3-3/TEAD1 were studied by immunoprecipitation and western blot. **b** Western blot showed total and phosphorylated protein of YAP and CTGF, a target gene of YAP in LOVO cells expressing GAS5-specific siRNA or SW620 cells expressing exogenous GAS5. **c** Western blot showed co-transfected with the YAP and GAS5 plasmid suppressed protein expression of CTGF, a target gene of YAP. **d** SW620 cells were transfected with YAP plasmids or co-transfected with GAS5 and subjected to a cycloheximide (CHX) chase assay. Immunoblot detection of YAP (left); IB data were quantified using the ImageJ software (right). After 24 h, CHX (10 μg/ml) was added to the cell culture medium, and incubation was continued for 0, 4, 8, 12, or 24 h. Error bars indicate the mean ± SD. ***P <0.001. **e** Ubiquitination assays of CRC cells co-transfected YAP with GAS5 plasmid (left) or GAS5-specific siRNA (right). The bottom panels depict the input of the cell lysates. Twenty-four hours after transfection, 10 nM MG132 was added to the 1640 culture medium and incubation was continued for 8 h. **f** SW620 cells were transfected with full length of GAS5 or each of the GAS5 mutants and analyzed with immunoblot. **g** The co-transfection of His-tagged YAP and exogenous GAS5 or each of the GAS5 mutants was immunoprecipitated with anti-His antibody and analyzed with immunoblot for ubiquitination. **h** Venn diagram and heatmap of the common differentially expressed genes after overexpression of GAS5 and YAP knockdown are shown. **i** Intersection analysis of KEGG pathway for differentially expressed genes in YAP knockdown and GAS5 upregulation groups (fold change ≥2; *p*-value < 0.05 in RNA-seq). **j** qRT-PCR analysis of cells with exogenous GAS5 or transfected with GAS5-specific siRNA compared with vector controls. Experiments were performed in triplicate, and data are presented as the mean ± SD. **P <0.01; and ***P <0.001

LncRNA GAS5 is localized both in the cytoplasm and the nucleus, while the majority is in the cytoplasm [13]. Moreover, the cytoplasmic localization of GAS5 promoted cytoplasmic retention of YAP by interacting with YAP. Immunoprecipitation assay indicated that GAS5 overexpression suppressed the interaction between YAP and TEA domain transcription factor 1 (TEAD1) but facilitated interactions between YAP and 14–3-3 or LATS1. Knockdown of GAS5 decreased YAP-14-3-3 or LATS1 interactions, but increased interactions between YAP and TEAD (Fig. 2a). It is noteworthy that knockdown of GAS5 decreased the level of YAP phosphorylation at serine 127 as expected and significantly up-regulated the expression of the YAP target gene CTGF. However, overexpression of GAS5 increased YAP phosphorylation and reduced CTGF expression (Fig. 2b). More interestingly, the total protein level of YAP was also regulated by GAS5, while the mRNA level of YAP was not changed by qRT-PCR analysis (Fig. 2j). Notably, western blot analysis showed that co-transfection with the YAP and GAS5 plasmid suppressed YAP and CTGF protein expression (Fig. 2c). Because GAS5 up-regulation significantly suppressed the total protein level of YAP while leaving the mRNA level of YAP unchanged, we performed cycloheximide (CHX) chase assays to detect whether GAS5 can attenuate YAP protein stability. Western blot analysis showed that the half-life of YAP protein was remarkably decreased to about 12 h in GAS5 overexpression cells, while the half-life in the control group was over 24 h (Fig. 2d). To further demonstrate whether GAS5 facilitates YAP protein ubiquitination and degradation, we treated CRC cells with proteasome inhibitor MG132. Ubiquitination assay showed a significant increase in poly-ubiquitinated YAP protein in GAS5-overexpressing cells, whereas YAP ubiquitination decreased in GAS5 knockdown cells (Fig. 2e). Interestingly, western blot analysis showed that overexpression of GAS5 mutant-Δ3 successfully promoted YAP phosphorylation as efficiently as full-length GAS5, whereas other mutants completely lost the capacity to promote YAP phosphorylation (Fig. 2f). Further ubiquitination assay showed that GAS5 mutant-Δ3 overexpression increased YAP ubiquitination, whereas other mutants had no effects on YAP ubiquitination (Fig. 2g). Remarkably, intersection analysis of KEGG pathway analysis for differentially expressed genes both in YAP knockdown and GAS5 up-regulation groups revealed a highly significant overlap of 543 common targets (FC > 2; *p*-value< 0.05), mostly deregulated in the same direction and enriched in the Hippo pathway, strongly supporting the key role of GAS5 in YAP signaling (Fig. 2h-i). Generally, these results demonstrated that GAS5 suppresses YAP signaling by promoting YAP phosphorylation and cytoplasmic sequestration to facilitate its ubiquitination and degradation.

### LncRNA GAS5 inhibits colorectal cancer progression via dysregulation of YAP in vitro and in vivo

**Fig. 3:**
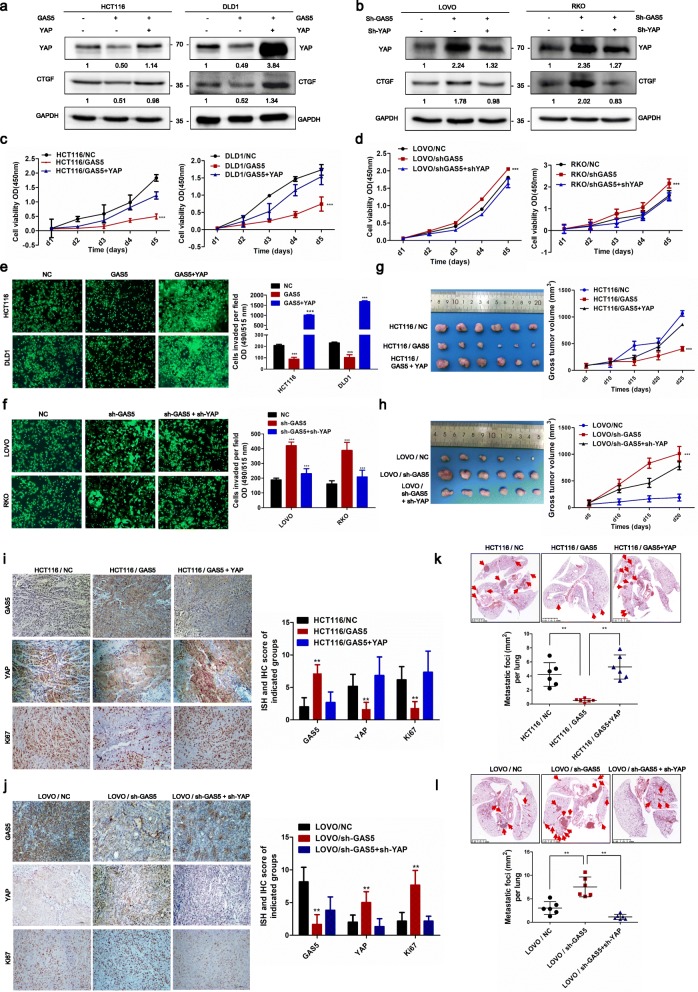
LncRNA GAS5 inhibits colorectal cancer progression via suppression of YAP in vitro and in vivo. **a** Co-transfected YAP with GAS5 reversed the GAS5-mediated decrease in CTGF expression in HCT116 and DLD1 cells, examined by western blot. **b** Knockdown of YAP inhibited the sh-GAS5-mediated up-regulation of CTGF in LOVO and RKO cells, examined by western blot. **c-d** CCK8 proliferation assays were performed to determine cell proliferation of CRC cells after co-transfection of GAS5 and YAP plasmid **(c)** or GAS5 and YAP specific shRNAs **(d)**. The mean ± SD is shown for five independent experiments. ***P <0.001. **e-f** Transwell assays were performed to investigate the changes in invasion abilities of CRC cells transfection, respectively. Transwell assays were quantified using the ImageJ software (right). All experiments were performed in triplicate, and results are presented as mean ± SD. ***P <0.001. **g-h** Representative images of tumors growth in xenografted BALB/c nude mice. Each group of mice were ectopically implanted with 2 X 10^6^ indicated cells into the flanks of mice (*n* = 6). Here, cells were transfected with indicated lentiviral vector or inducible shRNA. And the volume of tumors in individual mice was calculated using ImageJ software (right panel). Results are presented as mean ± SD. ***P <0.001. **i-j** Representative images of ISH and IHC staining on paraffin-embedded samples of xenograft tumors growth in BALB/c nude mice. **k-l** Representative lung tissues images of mice lung metastasis number and foci are shown by HE staining. And the area of metastases nodules in individual mice was calculated using Dmetrix software (bottom panel). (*n* = 6); **P <0.01

To investigate the role of GAS5 in CRC progression in vitro and in vivo, we constructed CRC cell lines stably overexpressing GAS5 or co-transfected with YAP. Western blot showed that overexpression of YAP significantly rescued the GAS5-mediated decreases in YAP and CTGF expression, whereas the knockdown of YAP greatly inhibited the sh-GAS5-mediated up-regulation of CTGF (Fig. 3a-b). Functional assays showed that overexpression of GAS5 significantly suppressed the proliferative and invasion capacity of CRC cells compared with that in control cells containing the empty vector, whereas increased YAP expression successfully reversed GAS5-mediated inhibition of CRC cell proliferation. Knockdown of GAS5 and YAP showed the opposite results (Fig. 3c–f).

Furthermore, in vivo assay demonstrates that overexpression of GAS5 significantly impaired tumor growth with a concomitant decline in cell viability and tumor volume compared with that in control group. Whereas overexpression of YAP resulted in dramatically accelerated tumor growth and increased tumor volume and significantly reversed the tumor suppression effect of GAS5 overexpression. Knockdown of GAS5 and YAP yielded the opposite results (Fig. 3g-h). Moreover, ISH and IHC staining on paraffin-embedded samples of xenograft tumors revealed that expression of YAP and Ki67 was significantly decreased in the xenograft tumors overexpressing GAS5 compared with that in the control group. Whereas, the expressions of YAP and Ki67 were greatly increased in the GAS5-silenced tumors compared with that in the control group (Fig. 3i-j). The mouse xenograft tumor model of lung metastasis by injecting the indicated cells into the tail veins of nude mice showed that GAS5-overexpressing cells demonstrated reduced lung colonization ability compared with the controls, whereas the co-expression of YAP abrogated the GAS5-mediated reduced lung colonization ability of CRC cells. Meanwhile, knockdown of GAS5 and YAP showed the opposite results (Fig. 3k-l). Generally, our data indicate that GAS5 represses CRC progression through suppression of YAP signaling in vitro and in vivo.

### YTHDF3 is a novel target of YAP and can be regulated by GAS5 in vitro and in vivo

**Fig. 4:**
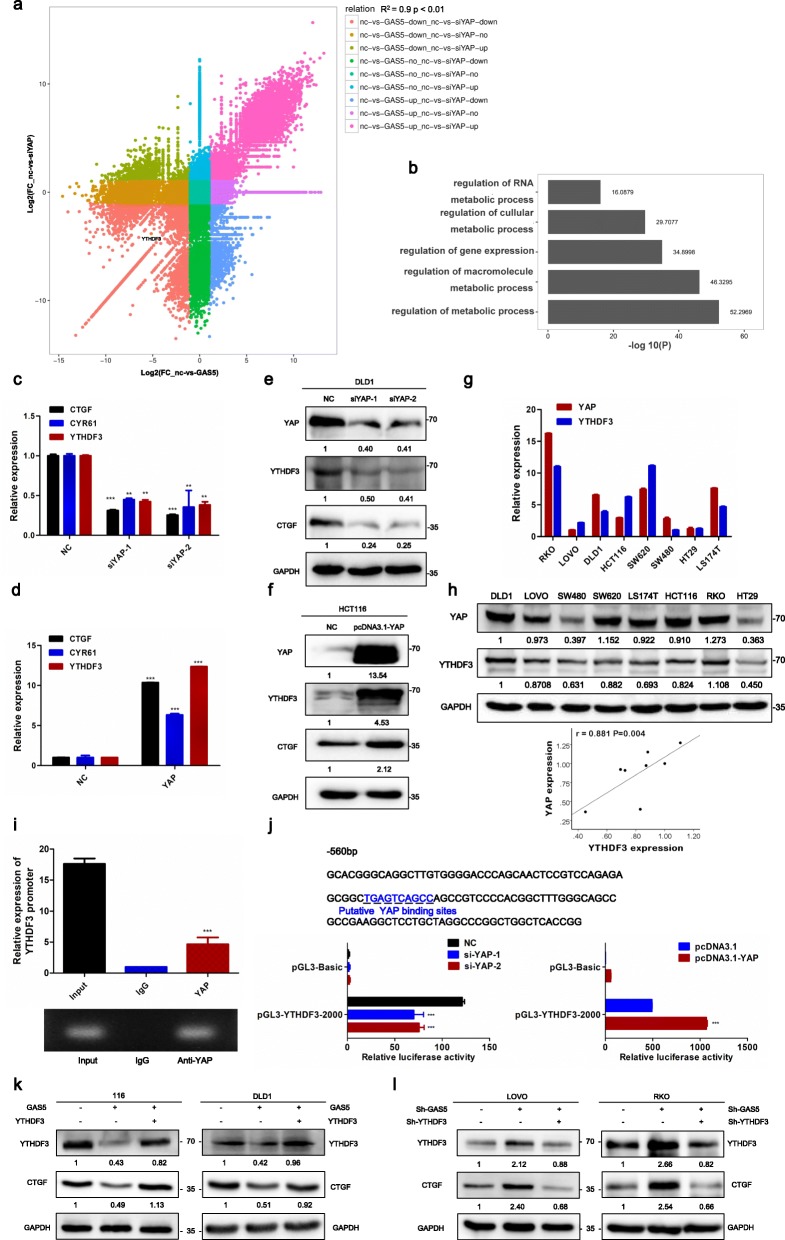
YTHDF3 is a novel target of YAP and can be regulated by GAS5. **a** The correlation of log2 fold change (FC) of differentially expressed genes in YAP knockdown cells and log2 (FC) of differentially expressed genes in GAS5 overexpressing cells are indicated. Genes were further stratified into groups based on the correlation between the YAP knockdown and GAS5 overexpression change. Group 1 (pink) were genes that were down-regulated in both treatment groups. Group 9 (purple) were those genes that were up-regulated in both treatment groups. (/log FC/ ≥ 1, *p*-value< 0.05 in RNA-seq). **b** Gene ontology (GO) analysis of differentially expressed genes (FC > 2; *p*-value < 0.05 in RNA-seq) in YAP knockdown and GAS5 up-regulation groups. **c-d** qRT-PCR detection of genes expressions of YAP target genes. All experiments were performed in triplicate, and results are presented as mean ± SD. **P < 0.01, and ***P < 0.001. **e-f** Analysis of the indicated protein expression in DLD1 cells transfected with YAP-specific siRNA or si-control **(e)**, and HCT116 cells transfected with YAP plasmid or vector **(f)**. **g-h** qRT-PCR and Western blots analysis showed a positively correlation between YAP and YTHDF3 in various CRC cells. **i** Binding of YAP to the YTHDF3 promoter was studied by chromatin immunoprecipitation (ChIP) assay. And the coprecipitated DNA was subjected for analysis of YTHDF3 by qRT-PCR (upper panel). The PCR procedures was shown by agarose gel electrophoresis (bottom panel). Experiments were performed in triplicate, and data are presented as mean ± SD. ***P < 0.001 **(j)** Potential YAP binding sites in the human YTHDF3 promoter between − 550 and + 100 bp. YAP plasmid or YAP-specific siRNA were transfected into HEK293 cells to detect the transcriptional activity of YTHDF3 promoter by dual luciferase reporter system. Experiments were performed in triplicate, and data are presented as mean ± SD. ***P< 0.001 **(k-l)** Analysis of the indicated protein expression in HCT116 and DLD1 cells co-transfected with GAS5 and YTHDF3 plasmid or YTHDF3 alone **(k)**, and LOVO and RKO cells co-transfected with sh-GAS5 and sh-YTHDF3 or sh-GAS5 alone **(l)**

Because de-regulation of YAP has significant implications for the pathobiology and progression of CRC, we performed RNA-interference-mediated knockdown of YAP to find potentially novel targets for CRC tumor progression. RNA sequencing analysis showed that hundreds of genes were changed in YAP-decreased cells when compared to the negative control. Interestingly, the correlation of differentially expressed genes in YAP knockdown cells and differentially expressed genes in GAS5 overexpressing cells showed a positive relationship between the treatment groups (r^2^ = 0.9; *P <* 0.01). The down-regulation of genes in the YAP knockdown group could be directly suppressed by the elevated expression of GAS5. Surprisingly, among these target genes of YAP, YTH-domain family member 3 (YTHDF3), a cytoplasmic N6-methyladenosine (m^6^A) reader, was consistently found to be down-regulated in both groups by more than 10-fold (Fig. 4a). Gene ontology enrichment analysis showed that the RNA metabolic process was significantly enriched in these differentially expressed genes (Fig. 4b), suggesting the key role of YAP in RNA modification. However, it remains largely unknown how YAP regulates m^6^A modification. To further answer this question, we performed qRT-PCR and western blot analysis and found that the overexpression of YAP increased YTHDF3 expression, as well as CTGF and CYR61 expressions, while knockdown of YAP significantly reduced expression of YTHDF3, CTGF and CYR61 (Fig. 4c–f). The mRNA and protein expressions showed positively correlations between YAP and YTHDF3 in CRC cells (Fig. 4g, h). Given the above results, we reasoned that YTHDF3 is a novel target of YAP. To further test this possibility, LASAGNA-Search software (2.0) was used to predict that the region from − 2000 to + 100 bp upstream of the YTHDF3 transcriptional start site contains YAP binding site (Fig. 4j). We further performed ChIP assays on paraformaldehyde cross-linked CRC cells to determine whether YAP binds to the YTHDF3 promoter in vitro. Results showed YAP binds to the promoter of YTHDF3 by ChIP-qPCR (Fig. 4i). To investigate the influence of YAP on YTHDF3 transcription, we measured the luciferase activities of YTHDF3 promoter vectors in the presence of YAP plasmid or YAP siRNA. YTHDF3 luciferase reporter assays indicated that the luciferase activity for YTHDF3 promoter was dramatically enhanced by YAP co-transfected into CRC cells, while the luciferase activity was significantly inhibited by YAP-specific siRNA (Fig. 4j).

As expected, gain-of-function and loss-of-function analysis indicated that YTHDF3 knockdown abrogated the YAP-mediated promotion of CRC cell proliferation and invasion, whereas YTHDF3 overexpression yielded the opposite results (Additional file 1: Figure S5). Moreover, western blot analysis showed that GAS5 overexpression suppressed expressions of YTHDF3 and CTGF, whereas YTHDF3 up-regulation reversed GAS5-mediated suppression of YTHDF3 and CTGF. Meanwhile knockdown of GAS5 and YTHDF3 yielded the opposite results (Fig. 4k-l).

**Fig. 5:**
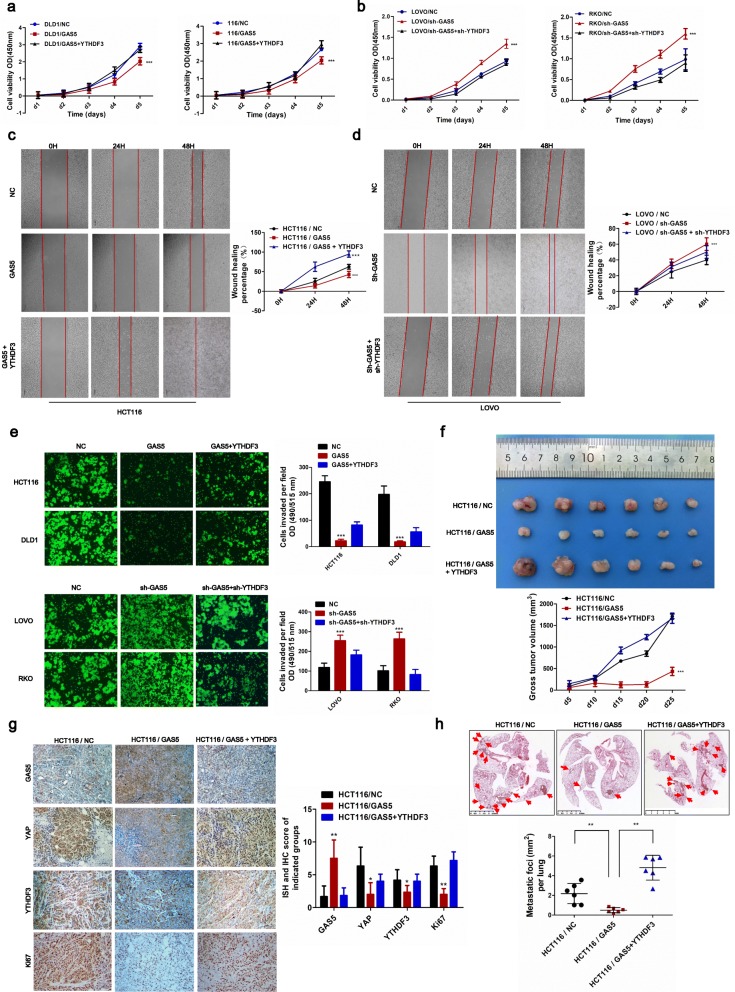
LncRNA GAS5 inhibits colorectal cancer progression via attenuation of YAP-mediated expression of YTHDF3 in vitro and in vivo*.* **a-b** CCK8 proliferation assays were performed to determine cell proliferation of CRC cells after co-transfection of GAS5 and YTHDF3 plasmid **(a)** or GAS5 and YTHDF3 specific shRNAs **(b)**. The mean ± SD is shown for five independent experiments. ***P < 0.001. **c-d** The scratch wound-healing assay were performed to investigate the changes in migratory abilities of HCT116 and LOVO cells after indicated transfection, respectively. The wound-healing percentage was analyzed with the ImageJ software (right). All experiments were performed in triplicate, and results are presented as mean ± SD. ***P < 0.001. **e** Transwell analysis assays were performed in the indicated CRC cells after transfection, respectively. Transwell analysis assays were quantified using the ImageJ software (right). All experiments were performed in triplicate, and results are presented as mean ± SD. ***P < 0.001 **(f)** Representative images of tumors growth in xenografted BALB/c nude mice. Each group of mice were ectopically implanted with 2 × 10^6^ indicated cells into the flanks of mice (*n* = 6). Here, cells were transfected with indicated lentiviral vector. Mice were sacrificed 25 days after implantation and tumor volume was measured (error bars represent mean ± SD). Scale bar, 1 cm. ***P < 0.001. **g** Representative images of ISH and IHC staining on paraffin-embedded samples of xenograft tumors growth in BALB/c nude mice. **h** Representative lung tissues images of lung metastasis model generated by injecting tumor cells into the tail veins of mice. HE staining showing the number and foci of lung metastases in each group (*n* = 6); **P < 0.01. And the area of metastases nodules in individual mice was calculated using Dmetrix software (bottom panel)

Functional assays showed that GAS5 up-regulation significantly suppressed the proliferative and invasive capacity of CRC cells, whereas co-transfection of YTHDF3 reversed the GAS5-mediated suppression of CRC cell proliferation. Knockdown of YTHDF3 yielded the opposite results (Fig. 5a-e). Furthermore, tumor subcutaneous growth and lung metastasis xenograft mice models demonstrated that overexpression of YTHDF3 resulted in dramatic acceleration of the tumor growth rate and lung colonization ability, which reversed the tumor suppression resulting from GAS5 overexpression (Fig. 5f and h). ISH and IHC staining on paraffin-embedded samples of xenograft tumors confirmed that GAS5 decreased YTHDF3 expression and that GAS5 overexpression decreased the expression of YAP and Ki67 in samples (Fig. 5g). Collectively, these data show that YTHDF3 is a novel target of YAP and GAS5 represses CRC cell proliferation and invasion through suppression of YAP-mediated expression of YTHDF3 in vitro and in vivo.

### YTHDF3 binds m^6^A modified GAS5 and promotes decay of GAS5

**Fig. 6:**
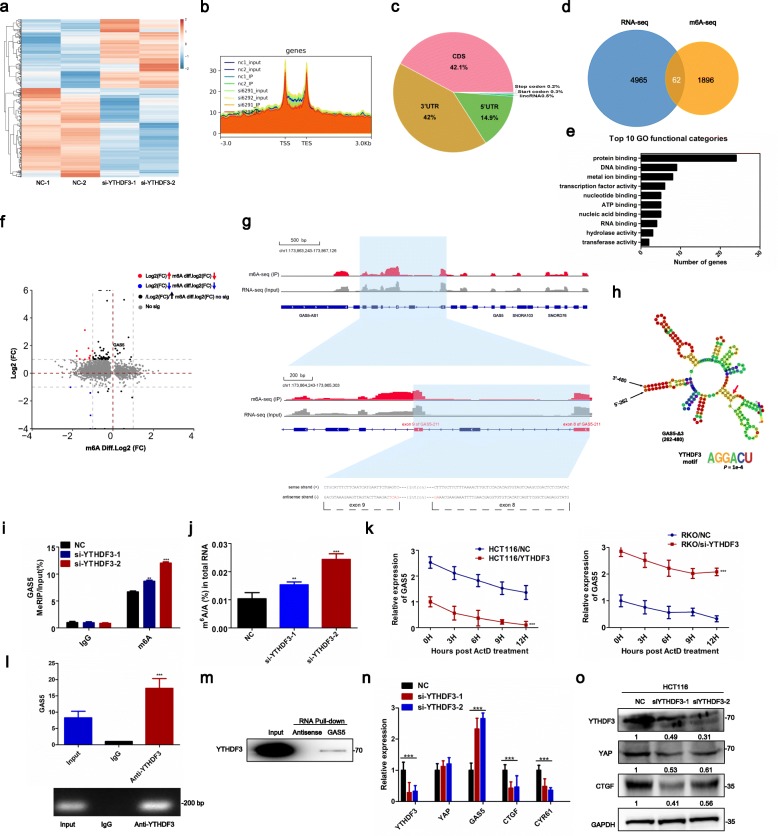
YTHDF3 binds m^6^A modified GAS5 and promotes its decay. **a** Heatmap showing the difference transcripts between normal and YTHDF3 knockdown groups. **b** Meta-gene analysis depicting different m^6^A peaks distribution between normal and YTHDF3 knockdown groups in a normalized transcripts. **c** Pie chart show the different m^6^A peaks distribution between normal and YTHDF3 knockdown groups in all transcripts. **d** A Venn diagram is used to display the common differentially expressed transcripts in RNA-seq and MeRIP-seq (/FC/> 2; *p*-value < 0.05). **e** Gene ontology (GO) analysis of differentially expressed genes both in YTHDF3 knockdown and m^6^A peaks groups (/FC/ > 2; p-value < 0.05). **f** The correlation of log2 (FC) of differentially expressed transcripts in YTHDF3 knockdown cells and log2 (FC) of differentially m^6^A modification transcripts in YTHDF3 knockdown cells are indicated (/log FC / ≥ 1; p-value < 0.05). Group 1 (red) were those transcripts that were up-regulated in RNA-seq, whereas declined in MeRIP-seq; Group 2 (blue) were transcripts that were down-regulated in both groups (/log FC/ ≥ 1, *p*-value< 0.05). **g** IGV analysis displayed the m^6^A peaks among GAS5 was located in exon 8 and exon 9. **h** Binding motif identified by MEME visualized YTHDF3 binding motif AGGACU among GAS5. **i** The m^6^A levels of GAS5 were quantified by m^6^A-RNA immunoprecipitation followed by qRT–PCR in HCT116 cells treated with Control or si-YTHDF3. All experiments were performed in triplicate, and results are presented as mean ± SD. **P < 0.01 and ***P < 0.001 **(j)** Global m^6^A RNA modification treated with control or si-YTHDF3 in HCT116 cells by the m^6^A RNA methylation quantification analysis. All experiments were performed in triplicate, and results are presented as mean ± SD. **P < 0.01 and ***P < 0.001. **k** qRT-PCR of GAS5 in actinomycin D-treated CRC cells. HCT116 cells were treated with YTHDF3 plasmid, while RKO cells were treated with siRNA targeting YTHDF3. Actinomycin D (100 nM, for 8 h) was used to inhibit transcription of the indicated gene. The mean ± SD is shown for five independent experiments. ***P < 0.001. **l** RIP <assays for YTHDF3 were performed and the co-precipitated RNA was subjected to qRT-PCR for GAS5 (upper panel). Agarose electrophoresis of PCR products (bottom panel). Experiments were performed in triplicate, and data are presented as mean ± SD. ***P < 0.001. **m** RNA pulldown assays and western blot assays showed that biotinylated-GAS5 could bind YTHDF3 in CRC cells in vitro. **n** qRT-PCR detection of indicated genes expression. All experiments were performed in triplicate, and results are presented as mean ± SD. ***P < 0.001. **o** Western blots showed that silencing of YTHDF3 decreased indicated genes expression

We found that GAS5 suppresses YAP-mediated transcription of YTHDF3 in CRC progression, whereas how YTHDF3 contributes to CRC progression remains unknown. Although the YTHDF protein can directly bind to and recognize m^6^A-modified mRNA, the biological function of YTHDFs in lncRNAs by a methylation-dependent mechanism is still unknown. We performed methylated RNA immunoprecipitation followed by sequencing (MeRIP–sequencing) combined with transcription sequencing to clarify the mechanism underlying YTHDF3 in CRC (Fig. 6a-b). Results showed that the exon region contains the highest percentage of m^6^A peaks, and lncRNAs contain approximately 0.5% of the total m^6^A modification (Fig. 6c). Venn diagram and gene ontology analysis of differentially expressed genes both in YTHDF3 knockdown and m^6^A peaks groups (/FC/> 2; *p*-value< 0.05) were used to identify the molecular function of YTHDF3 associated m^6^A modification genes, which showed that these genes were enriched for functions associated with protein binding (Fig. 6d-e). Most importantly and intriguingly, for YTHDF3 knockdown groups, nearly 90% (54 of 62) of transcripts were up-regulated in differential m^6^A modification transcripts, including up-regulation of GAS5 transcription (fold change ≥2 and *p*-value< 0.05) (Fig. 6f). As expected, we performed integrative genomics viewer analysis to verify that m^6^A peaks among GAS5 was located in exon 8 and exon 9, which contains YTHDF3 binding motif AGGACU (Fig. 6g-h). Sequenced MeRIP-PCR data showed that the m^6^A level of GAS5 was increased in YTHDF3-silenced CRC cells compared with that in the negative control (Fig. 6i).

We further detected the global m^6^A RNA modification in CRC cells using the m^6^A RNA Methylation Quantification Kit (abcam, MA, USA). Results showed that the total percentage of m^6^A in YTHDF3-silenced CRC cells increased two-fold compared with that in its negative control (Fig. 6j). Next, we performed RNA life-time profiling by incubation with transcription inhibitor actinomycin D on YTHDF3 overexpression or silenced CRC cells and obtained RNA at different time points. Indeed, YTHDF3 overexpression led to shortened lifetimes of GAS5 when compared with that in the negative control. Whereas, knockdown of YTHDF3 led to prolonged lifetimes of GAS5 in comparison with the siRNA control (Fig. 6k). Thus, YTHDF3 knockdown led to significantly increased expression of GAS5 as a result of accumulation of m^6^A modifications in GAS5, suggesting the primary role of YTHDF3 in m^6^A-mediated degradation of GAS5.

To test the interaction between YTHDF3 and GAS5, we performed RNA pull-down assays and subsequent western blot analyses to detect GAS5 interactions with YTHDF3 (Fig. 6m). Further RIP experiments, qRT-PCR, and agarose gel electrophoresis assays demonstrated that YTHDF3 directly bonded to GAS5 to form an m^6^A modification complex (Fig. 6l). Moreover, qRT-PCR and western blot indicated that both total expression of GAS5 and YAP target genes were elevated in YTHDF3-silenced CRC cells (Fig. 6n-o). In conclusion, here we show that YTHDF3 selectively binds to m^6^A-modifed GAS5 and modulates GAS5 degradation in a methylation-dependent manner, giving a basis for dysregulation of GAS5 in CRC progression.

### LncRNA GAS5 expressions negatively correlates with YAP and YTHDF3 protein levels in tumors from CRC patients

**Fig. 7:**
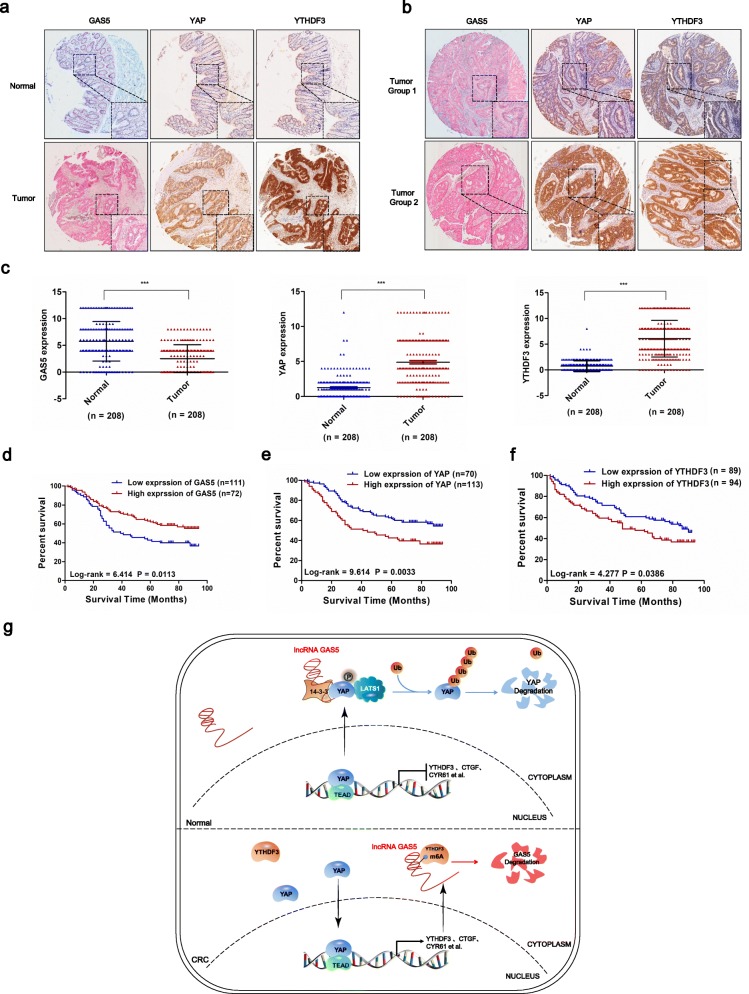
LncRNA GAS5 expression negatively correlates with YAP and YTHDF3 levels in CRC patient samples. **a-b** The ISH staining of GAS5 and IHC staining of YAP and YTHDF3 in tumor tissues and adjacent normal tissues of CRC paraffin-embedded samples. **c** The expression levels of GAS5, YAP and YTHDF3 in FFPE colon cancers and normal tissues were showed in indicated scattergram using ImageJ (*n* = 208). Data are shown as mean ± SD. ***P < 0.001. **d-f** Kaplan–Meier plot of overall survival of CRC patients with GAS5 **(d)**, YAP **(e)** and YTHDF3 expression **(f)**. **g** A schematic model for a negative feedback loop between YAP-binding lncRNA GAS5 and the m^6^A reader YTHDF3 in CRC tumor progression

Finally, we assessed the clinical expression of GAS5 and YAP or YTHDF3 in CRC by ISH and IHC staining on 208 paraffin-embedded CRC specimens from cohort 1. We found that expressions of GAS5 were lower in tumor tissues compared with their normal mucosal counterparts. Meanwhile, YAP and YTHDF3 protein levels were excessively elevated in most of the primary CRC tissues (Fig. 7a and c). Generally, higher expressions of GAS5 were usually accompanied by lower expressions of YAP and YTHDF3, while lower expressions of GAS5 were associated with elevated expressions of YAP and YTHDF3 in tumor tissues from CRC patients (Fig. 7b). In addition, correlations among the expression of GAS5, YAP, and YTHDF3 were analyzed with Spearman’s rank correlation. The scatter plot showed a negative relationship between GAS5 and YAP (r^2^ = − 0.3103; *P <* 0.001) and a positive relationship between YAP and YTHDF3 (r^2^ = 0.6451; *P <* 0.001) in 208 CRC specimens. As shown in Additional file 3: Table S1 statistical analysis represented a strong correlation between GAS5 expression and tumor metastasis (*P *= 0.040) and TNM stage (*P *= 0.038) in cohort 1. Notably, YAP and YTHDF3 expression were also significantly correlated with TNM stage (*P *= 0.037 for YAP and *P *= 0.031 for YTHDF3; Additional file 3: Tables S2 and S3). Furthermore, we analyzed the association between GAS5 and overall survival rates of CRC patients in cohort 2, which included 183 cases of CRC patients with clinical follow-up data. Seventy-two tumor tissue specimens (39.3%) exhibited a high expression of GAS5, and the other 111 cases (60.7%) had low expression. Furthermore, Kaplan–Meier analysis indicated that the lower GAS5 expression was related to poor overall survival in patients with CRC (log-rank = 6.414, *P *= 0.0113). The median survival time of the CRC patients with lower GAS5 expression was 40 months, which was significantly shorter than the survival time of those with higher GAS5 expression (72 months) (Fig. 7d). Kaplan–Meier and log-rank test analyses suggested a positive correlation between YAP expression and significantly reduced overall survival rates (Log-rank = 9.614, *P *= 0.0033; Fig. 7e). It was also observed that higher expression of YTHDF3 protein was a significant prognostic factor for poor overall survival in CRC patients (Log-rank = 4.277, *P *= 0.0386) using Kaplan–Meier analysis. The median survival time of CRC patients with higher YTHDF3 expression was about 48 months, which was significantly shorter than those with lower YTHDF3 expression (about 86 months) (Fig. 7f). Together, these results clearly show that the expressions of GAS5 are reduced in CRC tissues compared with adjacent tissues, and lower GAS5 expressions are associated with elevated expressions of YAP and YTHDF3 in tumor tissues from CRC patients. Thus, we found that higher expression of YTHDF3 is a significant prognostic factor for poor overall survival in CRC patients, offering a promising approach for CRC treatment.

## Discussion

YAP sequestered in the nucleus is essential for YAP-mediated target gene transcription and tumor progression. TEAD family transcription factors are major nuclear partners that effect transcriptional activation [21]. Serine/threonine-protein kinase LATS1, a core component of the Hippo pathway, is the key regulator of YAP phosphorylation and facilitates its cytoplasmic localization [22]. Therefore, targeting the Hippo-YAP pathway may provide new approaches for cancer therapy [4, 23, 24]. In addition to these, c-Abl, a 140-kDa proto-oncoprotein, directly phosphorylates YAP at position Y357 to stabilize YAP in a c-Abl kinase-dependent manner [25]. The β-catenin-YAP1-TBX5 transcriptional complex is reported to be essential for tumor survival and tumorigenesis [26]. YAP is O-GlcNAcylated by O-GlcNAc transferase at serine 109 in a LATS1-dependent manner [27]. The ARID1A-containing SWI/SNF complex inhibits YAP transcription activity by blocking the association between YAP and TEAD [28]. Non-coding RNAs, such as microRNAs and circular RNAs also have been reported to play vital roles in targeting YAP. For example, miR-15a and miR-16-1 directly bind with YAP1 3’UTR to regulate YAP1 expression [29]. CircFAT1 is reported to abundantly sponge miR-375 to up-regulate YAP1 expression [30]. To date, lncRNAs have been found to interact with the core components of the Hippo-YAP pathway at different levels. For example, a ROR1-HER3-LncRNA MAYA signaling axis was reported to modulate the Hippo-YAP pathway by methylating Hippo/MST1 at Lys59 [31]. A previous study showed that lncARSR binds with YAP to impede LATS1-induced YAP phosphorylation and facilitates YAP nuclear translocation in propagation of renal tumor-initiating cells [32]. Nevertheless, the key non-coding RNAs involved in YAP signaling, especially those that directly interact with YAP, remain largely unclear. Therefore, their functions in cancer progression, including CRC, are also not well-characterized.

Here, we identified several candidates for YAP-interacting lncRNAs that may be key regulators for YAP signaling. We established a systematic strategy to screen YAP-interacting lncRNA by RIP-seq, RNA pull-down, BLI analysis, RNA FISH, and immunofluorescence co-staining assays. At present, there is no systematical study to identify YAP-interacting lncRNAs. Using the interaction between lncRNA GAS5 and YAP as a model, we also developed a new method to assess binding affinity of lncRNAs and protein using BLI analysis. Although RNA pull-down is one of the most common methods for detecting the interaction between lncRNAs and proteins, it cannot evaluate the binding affinity of RNA and proteins quantitatively. BLI analysis has been widely used in compound screening, protein interaction, and virus titer analysis [33]. To the best of our knowledge, ours is the first study to use BLI analysis to investigate the binding affinity of lncRNAs and protein complexes. Our study indicated that BLI analysis may offer a promising approach for examining the binding affinity of the lncRNA-protein complex. Our method attempts to preserve the RNA hairpin structure as much as possible to delete GAS5 mutants, while enabling the analysis of interactions between GAS5 and YAP. In particular, we identified nucleotides 262–480 of GAS5, which directly bonded with the WW domain of YAP to facilitate its cytoplasmic retention. Despite numerous studies, the RNA structural domains and the functional role in RNA and protein interaction remain largely unknown. Structural analysis of the lncRNA-protein complex will be of great importance for illuminating the concise mechanism for YAP-GAS5 interactions.

Moreover, our study also revealed that GAS5 directly binds to YAP to trigger YAP phosphorylation at Ser127 in CRC cells, which helps YAP cytoplasmic localization and facilitates its ubiquitin-mediated degradation. Functional analyses and a mouse xenograft tumor model showed that overexpression of GAS5 significantly suppressed the proliferative and metastasis capacity of CRC cells, whereas the exogenous YAP expression could successfully reverse GAS5-mediated inhibition of CRC tumor progression in vitro and in vivo. A previous study showed that lncARSR binds with YAP to impede LATS1-induced YAP phosphorylation and facilitates YAP nuclear translocation in propagation of renal tumor-initiating cells [32]. Whether lncRNAs can contribute to YAP protein stability remains largely unclear. Here, our study demonstrated that GAS5 works as an RNA scaffold to promote degradation of YAP in CRC progression. Our data uncovered a key mechanism by which GAS5 inhibited CRC proliferation and metastasis through directly binding with YAP and facilitates its phosphorylation and subsequently ubiquitin-mediated degradation. In the absence of GAS5, YAP translocated in the nucleus and activated its target gene transcription, which in the context of CRC would promote a more malignant phenotype. It will be of great interest to identify common lncRNAs that regulate YAP protein stability.

More importantly, we identified YTHDF3 as a novel target of YAP, which plays a key role in CRC progression. YAP signaling is essential for cancer progression; therefore, the findings of the novel and key targets will be of great importance for understanding the role and the mechanisms of YAP pathway in cancer. In our study, YTHDF3 is significantly elevated in CRC tumor tissues compared with that in counterpart normal tissues. Functionally, gain-of-function and loss-of-function experiments showed that YAP significantly promoted the proliferation, invasion, and metastasis of CRC in vitro and in vivo. In contrast, YTHDF3 knockdown reversed YAP-mediated promotion of CRC tumor progression, and co-transfection of YTHDF3 and GAS5 also obtained similar results. Therefore, our data indicated that YTHDF3, as a novel target of YAP, plays a key role in CRC progression in vitro and in vivo, which may provide new insights into CRC therapy.

Ultimately, we established a key role of YTHDF3 in m^6^A-modified GAS5 and degradation of GAS5 transcription, uncovering a negative feedback loop between YAP-interacting lncRNA GAS5 and the m^6^A reader YTHDF3 in Hippo/YAP signaling and tumor progression of CRC. We found that YTHDF3 knockdown significantly prolonged the decay rate of GAS5 as a result of accumulation of m^6^A modifications in GAS5 and therefore decreased YAP protein expression. Numerous studies indicated that expression of GAS5 was repressed in many types of malignant tumors [34, 35]. Nevertheless, very little is known about its mechanism for downregulation or degradation. It has been suggested that GAS5 transcription may be degraded by nonsense surveillance, also known as the nonsense-mediated RNA decay pathway [36]. Interestingly, a recent study showed that lncRNA XIST is highly methylated by RBM15 and METTL3, which is required for XIST-mediated transcriptional repression [37]. This study suggested that METTL3-mediated m^6^A modification is important for lncRNA expression. Another study also indicated that the internal m^6^A modification of linc1281 is required for mESC differentiation [38]. In our study, we clarified one mechanism by which GAS5 decayed through m^6^A modification, suggesting a novel lncRNA regulatory mechanism. Extensive studies should be done to analyze m^6^A modification leading to lncRNA destabilization and shed light on future studies of YTHDF3.

## Conclusions

Collectively, our study uncovers a functional link between lncRNAs and the m^6^A modification in YAP signaling in CRC. LncRNA GAS5 directly binds with YAP to facilitate its phosphorylation and ubiquitin-mediated degradation and thereby attenuate YAP-mediated transcription of YTHDF3, which reversibly and selectively binds m^6^A-methylated GAS5 to trigger its decay and forms a negative feedback loop. Based on our findings, we suggest a negative functional loop of lncRNA GAS5-YAP-YTHDF3 axis in the progression of CRC, which may offer a promising approach for CRC treatment.

## Supplementary information

**Additional file 1: Figure S1.** Validation of YAP-interacting lncRNAs in CRC cell lines. **Figure S2.** Full-length of human lncRNA GAS5 gene cloning. **Figure S3.** RNA FISH and immunofluorescence co-staining showed co-localization of GAS5 and YAP in CRC cells. **Figure S4.** Secondary structure model of Full-length and a series of deletion mutants of GAS5 were demonstrated using RNAstructure software. **Figure S5.** Inhibition of YTHDF3 reversed YAP-mediated promoting of CRC progression.

**Additional file 2.** Antibodies, oligonucleotide sequences and primers for this study.

**Additional file 3.** Relationship between GAS5, YAP and YTHDF3 expression and the clinical characteristics of CRC patients from Cohort 1.

**Additional file 4: Supplementary raw data 1.** Identification of YAP-associated lncRNAs via RNA-binding protein immunoprecipitation sequencing (RIP-seq) experiments.

**Additional file 5: Supplementary raw data 2.** Analysis of lncRNA-seq-based differential expression after knockdown of YTHDF3.

**Additional file 6: Supplementary raw data 3.** Analysis of MeRIP-seq analysis combined with transcription sequencing to clarify the differential expression after knockdown of YTHDF3.

## Data Availability

Supplementary methods and materials, Figures S1 to S5, and Table S1 and S2 are attached.

## Abbreviations

AML: Acute myeloid leukemia
BLI: Bio-layer interferometry
ChIP: Chromatin immunoprecipitation
CHX: Cycloheximide
CRC: Colorectal cancer
FTO: Fat mass and obesity associated protein
GO: Gene ontology
H&E staining: Hematoxylin-eosin staining
IGV: Integrative Genomics Viewer
IHC: Immunohistochemistry
ISH staining: In Situ Hybridization staining
lncRNA: Long noncoding RNA
m^6^A: N6-methyladenosine
MeRIP-sequencing: Methylated rna immunoprecipitation followed by sequencing
mRNAs: Messenger rnas
NMD: Nonsense-mediated rna decay
NSB: Nonspecific binding
RIP-sequencing: RNA-binding protein immunoprecipitation sequencing
RNA FISH: RNA fluorescent in situ hybridization
RNA-sequencing: RNA sequencing
TSS: Transcriptional start site
YTHDF3: YTH-domain family member 3
