# Supplementary material for: Long noncoding RNA GAS5 inhibits progression of colorectal cancer by interacting with and triggering YAP phosphorylation and degradation and is negatively regulated by the m6A reader YTHDF3
Source: Mol Cancer. 2019 Oct 16;18:143. doi: 10.1186/s12943-019-1079-y (PMC6794841; doi:10.1186/s12943-019-1079-y)
Supplement: Supplementary file 1 — Additional file 1: Figure S1. Validation of YAP-interacting lncRNAs in CRC cell lines. Figure S2. Full-length of human lncRNA GAS5 gene cloning. Figure S3. RNA FISH and immunofluorescence co-staining showed co-localization of GAS5 and YAP in CRC cells. Figure S4. Secondary structure model of Full-length and a series of deletion mutants of GAS5 were demonstrated using RNAstructure software. Figure S5. Inhibition of YTHDF3 reversed YAP-mediated promoting of CRC progression. [file 12943_2019_1079_MOESM1_ESM.pdf]

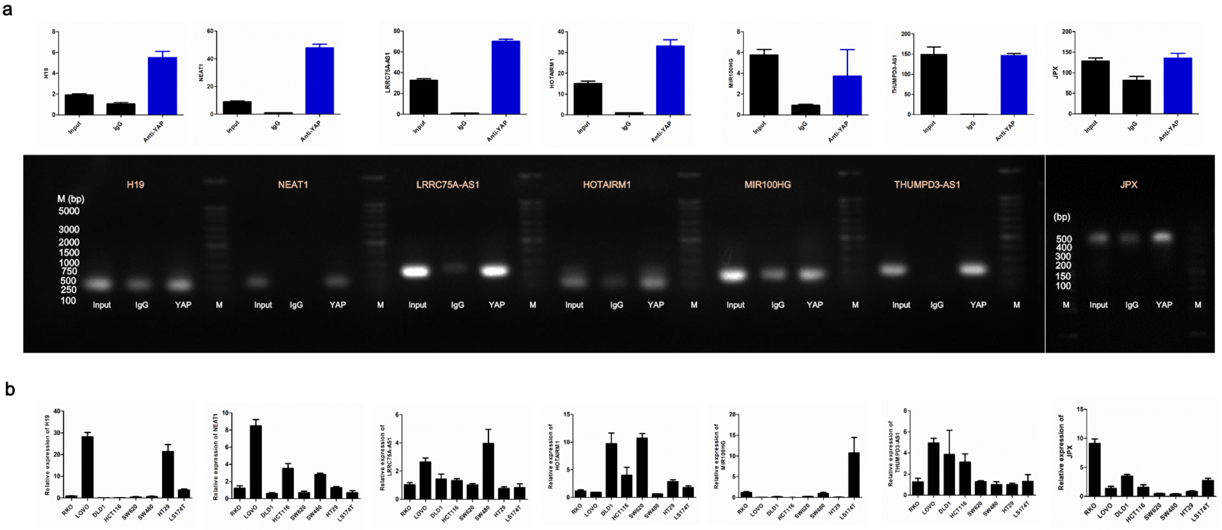

Figure S1, Validation of YAP-interacting lncRNAs in CRC cell lines. (a) RIP assays for YAP were performed and the top eight of cancer-related lncRNAs in RIP-seq was subjected to qRT-PCR (upper panel). Agarose electrophoresis of PCR products (bottom panel). Experiments were performed in triplicate, and data are presented as mean ± SD. ***P < 0.001. (b) The relative abundance of candidate lncRNAs were analyzed by qRT-PCR in various CRC cell lines. Related to Figure 1.

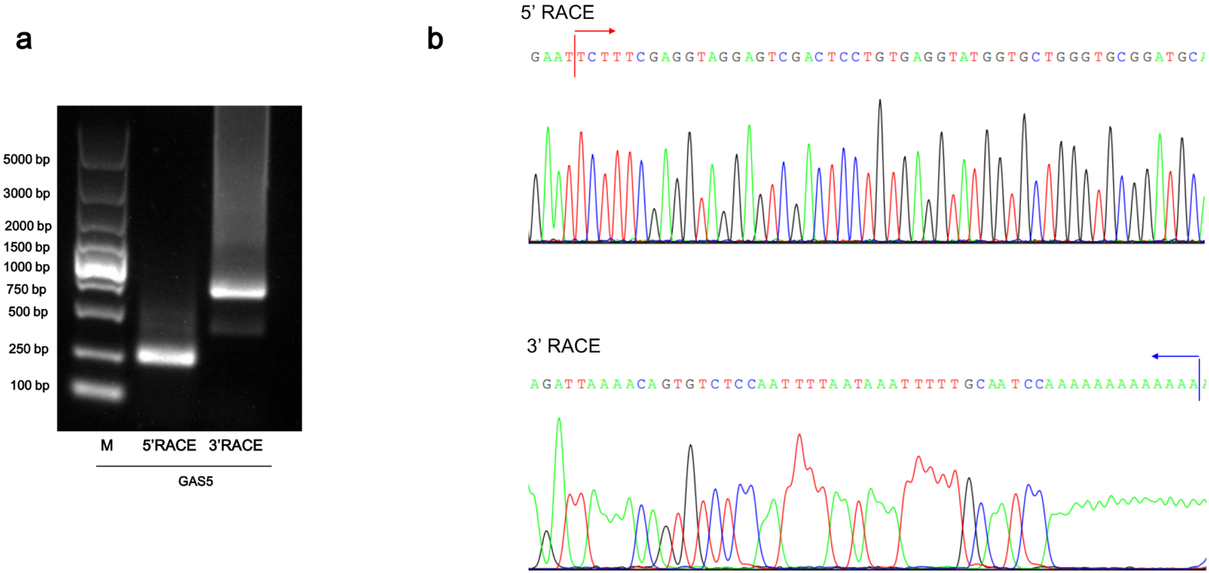

Figure S2, Full-length of human lncRNA GAS5 gene cloning. (a) PCR products from the 5′-RACE and 3′-RACE procedures as shown by agarose gel electrophoresis. (b) Nucleotide sequence of the full-length human lncRNA GAS5 gene.

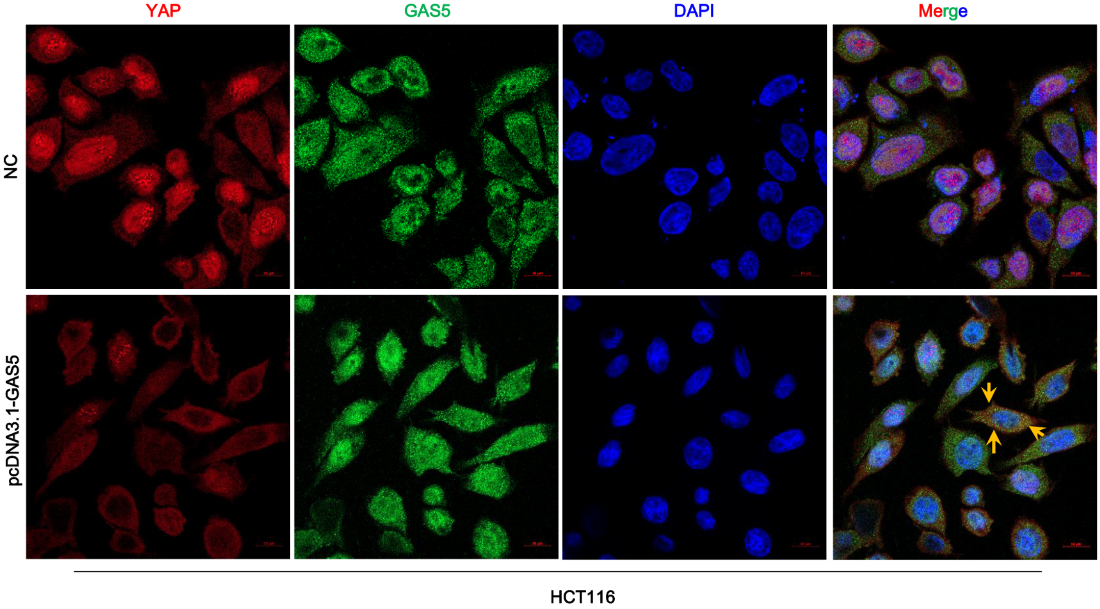

Figure S3, RNA FISH and immunofluorescence co-staining showed co-localization of GAS5 and YAP in CRC cells.

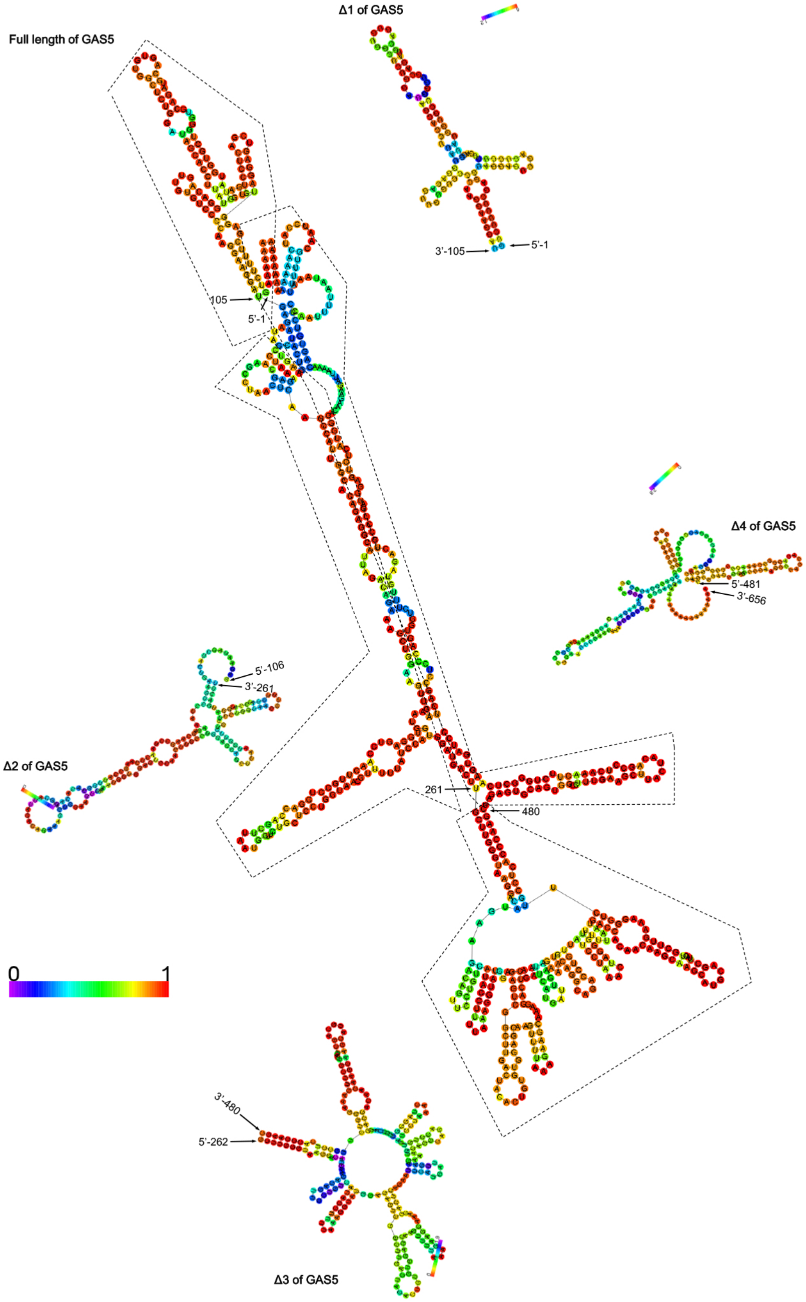

Figure S4, Secondary structure model of Full-length and a series of deletion mutants of GAS5 were demonstrated using RNAstructure software. Related to Figure 1.

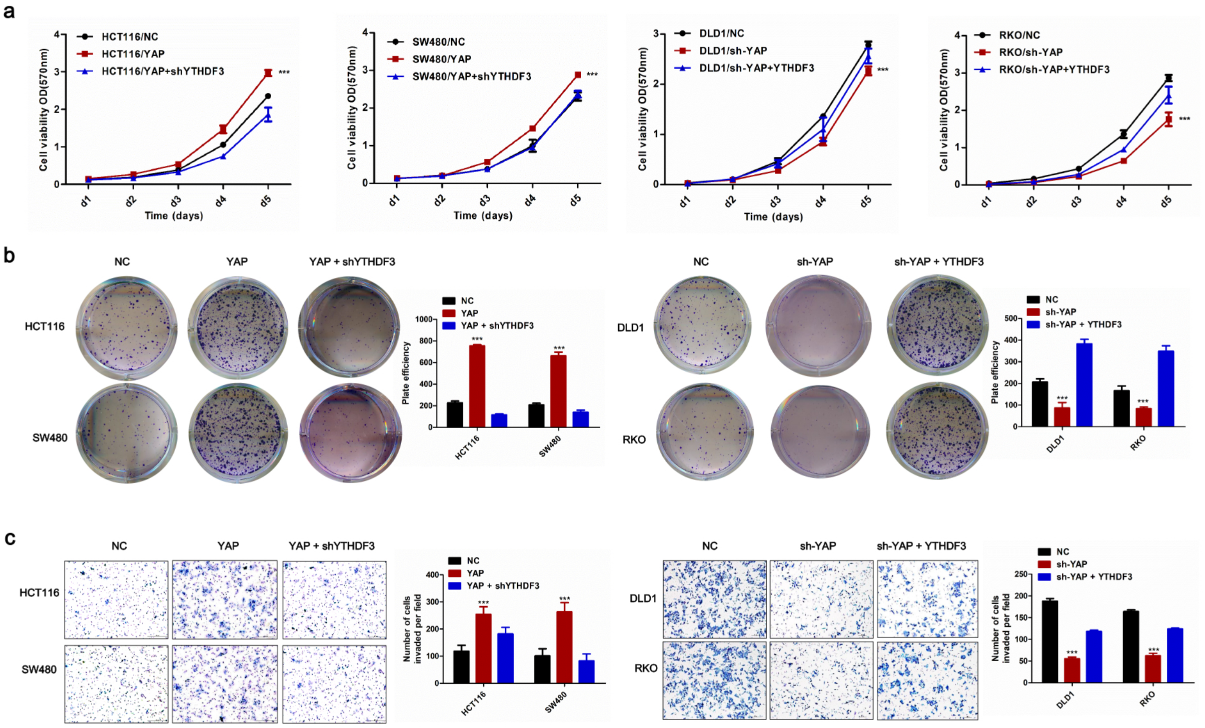

Figure S5, Inhibition of YTHDF3 reversed YAP-mediated promoting of CRC progression. (a) CCK8 proliferation assays were performed to determine cell proliferation of CRC cells after transfection of EGFP-LV-YAP or sh-YTHDF3, compared with control cell lines containing the empty vector. ***P < 0.001 (b) Colony formation assays were performed to determine cell proliferation of CRC cells after transfection of EGFP-LV-YAP or sh-YTHDF3, compared with control cell lines containing the empty vector. ***P < 0.001 (c) Transwell assays were performed to investigate the changes in migratory abilities of CRC cells transfection, respectively. Transwell assays were quantified using the ImageJ software (right). All experiments were performed in triplicate, and results are presented as mean ± SD. ***P < 0.001 Related to Figure 4.
