# Supplementary material for: Long noncoding RNA GAS5 inhibits progression of colorectal cancer by interacting with and triggering YAP phosphorylation and degradation and is negatively regulated by the m6A reader YTHDF3
Source: Mol Cancer. 2019 Oct 16;18:143. doi: 10.1186/s12943-019-1079-y (PMC6794841; doi:10.1186/s12943-019-1079-y)
Supplement: Supplementary file 2 — Additional file 2. Antibodies, oligonucleotide sequences and primers for this study. [file 12943_2019_1079_MOESM2_ESM.pdf]

Antibodies, oligonucleotide sequences and primers for this study

**Antibodies**	**SOURCE**	**SPECIES REACTIVITY**
YAP (D8H1X) XP® rabbit mAb	Cell Signaling Technology	Human and mouse
Rabbit monoclonal Anti-YAP1 antibody [EPR19812] (Alexa Fluor® 647)	abcam	Human and mouse
GFP (4B10) mouse mAb	Cell Signaling Technology	Human
LATS1 (C66B5) rabbit mAb	Cell Signaling Technology	Human
Rabbit anti-14-3-3 (pan)	Cell Signaling Technology	Human
TEAD1 (D9X2L) rabbit mAb	Cell Signaling Technology	Human
Phospho-YAP (Ser127) (D9W2I) rabbit mAb	Cell Signaling Technology	Human and mouse
CTGF (D8Z8U) rabbit mAb	Cell Signaling Technology	Human
Ubiquitin (P4D1) mouse mAb	Cell Signaling Technology	Human
Rabbit polyclonal anti-YTHDF3	abcam	Human and mouse
Mouse monoclonal anti-Lamin B1	Proteintech	Human
Mouse polyclonal anti-Ki67	ZSGB-BIO	Human and mouse
**Chemicals, Peptides, and Recombinant Proteins**	**SOURCE**	**IDENTIFIER**
Recombinant Human YAP1 protein	abcam	Cat#ab132459
Cycloheximide	MedChemExpress	Cat#HY-12320
MG-132	Selleck	Cat#133407-82-6
Actinomycin D	MedChemExpress	Cat#HY-17559
**Critical Commercial Assays**	**SOURCE**	**IDENTIFIER**
Magna RIP™RNA-Binding Protein Immunoprecipitation Kit	Millipore	Cat# 17-701
SMARTer® RACE 5'/3' Kit	Clontech	Cat# 634858, 634859
DIG Northern Starter Kit	Roche	Cat# 12 039 672 910
PARIS™ Kit	Invitrogen	Cat# AM1921
SimpleChIP® Enzymatic Chromatin IP Kit (Magnetic Beads)	Cell Signaling Technology	Cat#9003
Dual-Glo® Luciferase Assay System	Promega	Cat#E2920
m6A RNA Methylation Assay Kit (Colorimetric)	abcam	Cat# ab185912
Magna MeRIP™ m6A Kit	Millipore	Cat# 17-10499
**Oligonucleotides**		

LNA™ ISH probe: GAS5	/5DigN/AAGCTGCATGCTTGCTTGTTGT /3Dig_N/	
**Primer name (for qRT-PCR)**	**Forward primer (5'-3')**	**Reverse primer (5'-3')**
YAP1 (NM_006106.5)	TAGCCCTGCGTAGCCAGTTA	TCATGCTTAGTCCACTGTCTGT
GAS5 (NR_002578.3)	TATGGTGCTGGGTGCGGAT	CCAATGGCTTGAGTTAGGCTT
YTHDF3 (NM_001277813.1)	TCAGAGTAACAGCTATCCACCA	GGTTGTCAGATATGGCATAGGCT
CTGF (NM_001901.3)	CAGCATGGACGTTCGTCTG	AACCACGGTTTGGTCCTTGG
CYR61 (NM_001554.5)	CTCGCCTTAGTCGTCACCC	CGCCGAAGTTGCATTCCAG
GAPDH (NM_002046.7)	AGCTGAACGGGAAGCTCACT	TGCTTAGCCAAATTCGTTG
**siRNA**	**Forward**	**Reverse**
si-YAP1-1	GGUCAGAGAUACUUCUUAAAU	UUAAGAAGUAUCUCUGACCAG
si-YAP1-2	GGUGAUACUAUCAACCAAAGC	UUUGGUUGAUAGUAUCACCUG
si-GAS5-1	GCUCUGGAUAGCACCUUAUTT	AUAAGGUGCUAUCCAGAGCTT
si-GAS5-2	CCAUUGGCACACAGGCAUUTT	AAUGCCUGUGUGCCAAUGGTT
si-YTHDF3-1	GGUGGAUUUCACCAGUUAAUG	UUAACUGGUGAAAUCCACCAA
si-YTHDF3-2	AGAUGGUGUAUUUAGUCAACC	UUGACUAAAUACACCAUCUGG
**Recombinant DNA**		
pcDNA3.1（+）-YAP1-6xhis	Shanghai Integrated Biotech Solutions Co.,Ltd	Lot No. M25539
pCDH-MSCV-MCS-EF1-GFP-puro-YAP1	Shanghai Integrated Biotech Solutions Co.,Ltd	Lot No. Y42526
pcDNA3.1（+）-GAS5	Shanghai Integrated Biotech Solutions Co.,Ltd	Lot No. GB160612-K79827
LV6-GAS5	Shanghai Integrated Biotech Solutions Co.,Ltd	Lot No. 180607EZ
pCDH-CMV-MCS-EF1-Puro-YTHDF3	Shanghai Integrated Biotech Solutions Co.,Ltd	Cat. # CD510B-1
