# Supplementary material for: Long noncoding RNA GAS5 inhibits progression of colorectal cancer by interacting with and triggering YAP phosphorylation and degradation and is negatively regulated by the m6A reader YTHDF3
Source: Mol Cancer. 2019 Oct 16;18:143. doi: 10.1186/s12943-019-1079-y (PMC6794841; doi:10.1186/s12943-019-1079-y)
Supplement: Supplementary file 3 — Additional file 3. Relationship between GAS5, YAP and YTHDF3 expression and the clinical characteristics of CRC patients from Cohort 1. [file 12943_2019_1079_MOESM3_ESM.pdf]

Table S1. Relationship between lncRNA GAS5 expression and the clinical characteristics of CRC patients from Cohort 1

**Features**	**n**	**High**	**Low**	**x^2**	***p*-value**
All cases	208	60	148		
Age				0.440	0.495
< 50	2	1	1		
≥50	206	59	147		
Gender				0.436	0.528
Man	132	36	96		
Female	76	24	52		
Histological grade				0.872	0.647
Well	1	0	1		
Moderately	155	43	112		
Poorly	52	17	35		
Tumor size(cm)				0.615	0.534
<5	85	22	63		
≥5	123	38	85		
Tumor metastasis				4.583	**0.040***
Negative	129	44	85		
Positive	79	16	63		
TNM stage				4.525	**0.038***
I and II	74	28	46		
III and IV	134	32	102		

**p* value < 0.05 was considered to indicate statistical significance. The *p* values were calculated in SPSS 19.0 using Pearson's chi-square test. Median score of GAS5 was used as cut-off value for analysis.

Table S2. Relationship between YAP expression and the clinical characteristics of CRC patients from Cohort 1

**Features**	**n**	**High**	**Low**	**x^2**	***p*-value**
All cases	208	80	128		
Age				0.114	1.000
< 50	2	1	1		
≥50	206	79	127		
Gender				0.672	0.460
Man	132	48	84		
Female	76	32	44		
Histological grade				1.543	0.462
Well	1	0	1		
Moderately	155	57	98		
Poorly	52	23	29		
Tumor size(cm)				0.448	0.563
<5	85	35	50		
≥5	123	45	78		
Tumor metastasis				2.719	0.108
Negative	129	44	85		
Positive	79	36	43		
TNM stage				4.934	**0.037***
I and II	74	21	53		
III and IV	134	59	75		

**p* value < 0.05 was considered to indicate statistical significance. The *p* values were calculated in SPSS 19.0 using Pearson's chi-square test. Median score of YAP was used as cut-off value for analysis.

Table S3. Relationship between YTHDF3 expression and the clinical characteristics of CRC patients from Cohort 1

**Features**	**n**	**High**	**Low**	**x^2**	***p*-value**
All cases	208	97	111		
Age				2.311	0.216
< 50	2	2	0		
≥50	206	95	111		
Gender				0.016	1.000
Man	132	62	70		
Female	76	35	41		
Histological grade				0.892	0.640
Well	1	0	1		
Moderately	155	73	82		
Poorly	52	24	28		
Tumor size(cm)				0.557	0.482
<5	85	37	48		
≥5	123	60	63		
Tumor metastasis				0.818	0.393
Negative	129	57	72		
Positive	79	40	39		
TNM stage				4.753	**0.031***
I and II	74	27	47		
III and IV	134	70	64		

**p* value < 0.05 was considered to indicate statistical significance. The *p* values were calculated in SPSS 19.0 using Pearson’s chi-square test. Median score of YTHDF3 was used as cut-off value for analysis.
